# Childhood body mass index trajectories: modeling, characterizing, pairwise correlations and socio-demographic predictors of trajectory characteristics

**DOI:** 10.1186/1471-2288-12-38

**Published:** 2012-03-29

**Authors:** Xiaozhong Wen, Ken Kleinman, Matthew W Gillman, Sheryl L Rifas-Shiman, Elsie M Taveras

**Affiliations:** 1 Obesity Prevention Program, Department of Population Medicine, Harvard Medical School and Harvard Pilgrim Health Care Institute, Boston, MA 02215, USA

## Abstract

**Background:**

Modeling childhood body mass index (BMI) trajectories, versus estimating change in BMI between specific ages, may improve prediction of later body-size-related outcomes. Prior studies of BMI trajectories are limited by restricted age periods and insufficient use of trajectory information.

**Methods:**

Among 3,289 children seen at 81,550 pediatric well-child visits from infancy to 18 years between 1980 and 2008, we fit individual BMI trajectories using mixed effect models with fractional polynomial functions. From each child's fitted trajectory, we estimated age and BMI at infancy peak and adiposity rebound, and velocity and area under curve between 1 week, infancy peak, adiposity rebound, and 18 years.

**Results:**

Among boys, mean (SD) ages at infancy BMI peak and adiposity rebound were 7.2 (0.9) and 49.2 (11.9) months, respectively. Among girls, mean (SD) ages at infancy BMI peak and adiposity rebound were 7.4 (1.1) and 46.8 (11.0) months, respectively. Ages at infancy peak and adiposity rebound were weakly inversely correlated (r = -0.09). BMI at infancy peak and adiposity rebound were positively correlated (r = 0.76). Blacks had earlier adiposity rebound and greater velocity from adiposity rebound to 18 years of age than whites. Higher birth weight z-score predicted earlier adiposity rebound and higher BMI at infancy peak and adiposity rebound. BMI trajectories did not differ by birth year or type of health insurance, after adjusting for other socio-demographics and birth weight z-score.

**Conclusions:**

Childhood BMI trajectory characteristics are informative in describing childhood body mass changes and can be estimated conveniently. Future research should evaluate associations of these novel BMI trajectory characteristics with adult outcomes.

## Background

Childhood body mass index (BMI) predicts adulthood obesity [1,2] and other long-term health outcomes [3-5]. But previous studies have observed weak or moderate correlations (r = 0.2-0.5) between early childhood (< 7 years of age) and adulthood BMI [6,7]. Most of these studies [2,8-10] have used BMI at fixed ages or change in BMI between fixed ages as predictors. This fixed-age approach assumes that individuals in the sample belong to a homogeneous group with similar developmental patterns, which seems unrealistic for childhood BMI [11]. Also, the biological meaning of childhood BMI at a given fixed age may differ among children who have different growth patterns (initiation, velocity, duration, etc.) in bone, muscle, and fat tissues. Instead, a more appealing way of examining childhood BMI is to model individual trajectories based on repeated BMI measures throughout childhood. The capacity of childhood BMI to predict adult BMI can potentially be improved by using a child's BMI trajectory, in addition to or in place of his or her BMI at specific ages.

Individual- and group-based approaches are the two distinct methods for studying childhood BMI trajectories in the literature. The group-based approach tries to generate several groups or classes that share overall patterns of changes in BMI [12], BMI z-score [13], or risk of high BMI [11] across childhood, using methods such as latent growth mixture modeling. Despite its simplicity in summarizing overall patterns, the group-based approach requires the investigator's subjective decisions on the number of groups, even after optimization by statistical software. It is also subject to arbitrary names or definitions of selected groups, substantial variations in patterns within each group, and un-satisfying generalizability (e.g., the number and patterns of groups often change among new samples). Alternatively, the individual-based approach examines the specific trajectory for each child and then estimates informative BMI characteristics, and thus allows for further links to individual-specific exposures or health outcomes. For example, from individual-specific trajectories, one can identify BMI milestones including infancy peak and adiposity rebound [14-17], and also estimate some novel features of BMI change, such as velocity and the area under a BMI trajectory curve. Modeling childhood BMI trajectory may reveal stronger ties between childhood and adulthood BMI, leading to a better rationale for childhood interventions to prevent obesity and other health outcomes in adulthood. However, previous studies using the individual-based approach are limited by restricted age periods, such as from birth to 3 years [14] or from 2 to 18 years [17,18]. Consequently, the full picture on correlations between BMI milestones throughout childhood remains unclear [14], as does their independent and interactive impacts on long-term outcomes.

Our aims are: 1) to build parametric models to fit BMI trajectory throughout childhood; 2) to estimate BMI trajectory milestones and related characteristics; and 3) to examine pairwise correlations and socio-demographic predictors of BMI trajectory characteristics.

## Methods

### Study sample

As part of the Collecting Electronic Nutrition Trajectory Data Using e-Records of Youth (CENTURY) Study, we extracted length/height, weight, and demographic data from electronic medical records of well-child visits from 1980 through 2008 at Harvard Vanguard Medical Associates (HVMA), a multi-site group practice in eastern Massachusetts. Details of the data collection methods can be found elsewhere [19]. The study protocol was approved by the Institutional Review Board of Harvard Pilgrim Health Care.

#### Inclusion criteria

In this analysis, to assure sufficient data points for accurately estimating individual-specific BMI trajectories, we included children who had their weight and length/height measured at a minimum of 18 visits between 1 week and 18 years. Specifically, we included children who had at least two visits during the age interval 1 week-2.9 months, two visits during 3-7.4 months, two visits during 7.5-13.4 months, two visits during 13.5-20.9 months, one visit during 21.0-29.9 months, one visit during 2.5-3.4 years, one visit during 3.5-4.4 years, one visit during 4.5-5.4 years, one visit during 5.5-6.4 years, three visits during 6.5-10.4 years, one visit during 10.5-14.4 years, and one visit during 14.5-18.0 years. We determined these age intervals and corresponding minimum numbers of visits based on the need for more data points during periods of fast change and around turning points [20], as well as on schedules of preventive pediatric health care recommended by the American Academy of Pediatrics [21]. To be eligible, children must therefore have been born between October 1, 1979 (and be 2.9 months on January 1, 1980, the first date of data extraction) and June 30, 1994 (and be 14.5 years old on December 31^st^, 2008, at the end of data extraction). These criteria limited our eligible sample to 142,346 children with 1,075,237 visits. Among them, 3,289 children (2.3%) with 81,550 visits (7.6%) met our criteria for minimum number and timing of visits. To assess potential selection bias, we compared demographics and birth characteristics of the analytic sample to the excluded age-eligible sample (139,057 children with 993,687 visits). There were no substantial differences in sex, birth weight, or year of birth between the two samples, but the analytic sample contained a higher proportion of whites (71.8% vs 42.9%) and a lower proportion of unknown race/ethnicity (15.3% vs 37.7%) as well as lower proportion (3.9% vs 5.2%) of Medicaid-insured children than the excluded sample (Table 1).

**Table 1:** Characteristics of the analytic and excluded age-eligible sample born between October 1, 1979 and June 30, 1994

Characteristic	Analytic sample	Excluded sample
***Child-level***		

**Total # of children**	3289	139057

**Sex, n (%)**		

Boys	1680 (51.1)	70216 (50.5)

Girls	1609 (48.9)	68841 (49.5)

**Race/ethnicity, n (%)**		

White	2362 (71.8)	59644 (42.9)

Black	214 (6.5)	14341 (10.3)

Other	168 (5.1)	7847 (5.6)

Unknown	503 (15.3)	52443 (37.7)

**Year of birth, n (%)**		

1979 ~ 1984	382 (11.6)	38343 (27.6)

1985 ~ 1989	1315 (40.0)	48075 (34.6)

1990 ~ 1994	1592 (48.4)	52639 (37.9)

**Birth weight in grams, mean (SD)**	3442 (488)	3433 (507)

**Type of health insurance, %**		

Medicaid	129 (3.9)	7,256 (5.2)

Non-Medicaid	3160 (96.1)	131801 (94.8)

***Visit-level***		

**Total # of visits**	81550	993687

**Age at visit (years), n (%)**		

0 ~ 1	25188 (30.9)	294540 (29.6)

2 ~ 5	17501 (21.5)	215744 (21.7)

6 ~ 10	16681 (20.5)	175422 (17.7)

11 ~ 14	12974 (15.9)	164369 (16.5)

15 ~ 18	9206 (11.3)	143612 (14.5)

SD, standard deviation

### Measures

At well-child visits, medical assistants measured children's weight and length/height according to the written protocol of HVMA. Anthropometric equipment is calibrated annually at HVMA, and a master trainer conducts periodic quality checks of anthropometric measures by medical assistants. Using pediatric scales, medical assistants measured weight without heavy clothes and shoes, and rounded it to the nearest 0.25 pound (0.11 kg). Although the position for length measure was not documented in medical records, medical assistants usually measured length without shoes in recumbent position using a paper-and-pencil technique (see below) for children younger than 24 months, and height without shoes in standing position for those aged 24 months or older [22].

Briefly, for the paper-and-pencil technique, the child lay supine on a piece of paper atop an examination table. The medical assistant drew a tick mark abutting the top of the child's head, and then straightened the child's legs, flattened the child's knees, flexed the child's foot to be perpendicular to the table, and marked the paper again at the bottom of the child's heels. The medical assistant then measured the distance between the two marks with a flexible tape, and rounded it to the nearest quarter inch. However, in our previous validation study among 0 to 24 month-old infants conducted at one of the participating pediatric practice sites, we found that the paper-and-pencil method systematically overestimated children's length compared with a reference method [22]. We converted our paper-and-pencil lengths to 0.953 × length measured by paper-and-pencil method + 1.8 cm, as estimated in the validation study [22]. We applied this regression correction for all children younger than 24 months, and recognize that this universal correction might artificially introduce some errors in a small number of children who were measured in standing position before 24 months. We calculated BMI as, weight in kilograms/(height or length in meters)^2^.

We extracted children's race/ethnicity from medical records, and then recoded it as non-Hispanic white, non-Hispanic black, or other race/ethnicity including Hispanic, Asian American, Native American, Alaskan Native, and Native Hawaiian or other Pacific Islander. We calculated internal z-score of birth weight as, (individual birth weight - mean value)/standard deviation, for boys and girls separately within the analytic sample. The type of health insurance, Medicaid vs. non-Medicaid, was retrieved from medical records.

### Statistical analysis

We chose ages 3 months, 6 months, 1 year, 3 years, 4 years, 7 years, 11 years, and 18 years to check the normality of age-specific BMI distribution. Q-Q plots and Kolmogorov-Smirnov tests showed that BMI was approximately normally distributed at most of these age points, except for some right skewness at 18 years of age (skewness, 0.86 for boys and 0.90 for girls). So the normality assumption for age-specific BMI distribution is fairly acceptable in this sample.

We performed the main data analysis in three steps: modeling BMI trajectory, estimating trajectory characteristics, and examining correlations and predictors of trajectory characteristics. Given the well-known sex differences [23] in childhood growth, we conducted steps 1 and 2 among boys and girls separately.

#### Step I

We used a fractional polynomial approach to model childhood BMI trajectory as a function of age [24,25]. Briefly, the expected value of BMI was modeled as $E(BMI)=b_{0}+\sum_{j=1}^{m}b_{j}Age^{p_{j}}$, where *m *is the degree of the model, and powers *p_j _*are selected from a fixed set of 8 candidate values, including -2, -1, -0.5, 0 or log, 0.5, 1, 2, and 3. To enhance the model interpretability and also reduce computational burden, we simplified the original fractional polynomial method by excluding duplicated powers. Since most children had two milestones or turning points, infancy peak and adiposity rebound, we set the minimum model degree *m *= 3. Accordingly, we considered 219 candidate models, including 56 models of 3^rd^-degree, 70 models of 4^th^-degree, 56 models of 5^th^-degree, 28 models of 6^th^-degree, 8 models of 7^th^-degree, and 1 model of 8^th^-degree (Table 2).

**Table 2:** Mixed effect models with the best fractional polynomial function for childhood BMI trajectory, by model degree^a^

Degree	No. of candidate models	Included age terms in the mixed effect model with the best fractional polynomial function								Goodness of fit (smaller is better)^b^	
	
		Age^(-2)^	Age^(-1)^	Age^(-0.5)^	log(Age)	Age^0.5^	Age	Age^2^	Age^3^	-2 Log likelihood	BIC
**Boys (N = 1,680)**											

3 rd degree	56				×	×	×			150196	150218

4th degree	70		×		×	×	×			149688	149710

5th degree	56	×	×	×	×	×				**147836**	**147858**

6th degree	28	×	×	×	×	×	×			148889	148911

7th degree	8	×	×	×	×	×	×	×		161668	161690

8th degree	1	×	×	×	×	×	×	×	×	166173	166181

**Girls (N = 1,609)**											

3 rd degree	56				×	×	×			141787	141809

4th degree	70		×	×	×	×				139990	140012

5th degree	56	×	×	×	×	×				**138131**	**138153**

6th degree	28	×	×	×	×	×	×			140079	140101

7th degree	8	×	×	×	×	×	×	×		152402	152424

8th degree	1	×	×	×	×	×	×	×	×	156241	156248

BIC, Bayesian information criterion

^a ^Data from 3,289 children with at least 18 well-child visits from age 1 week to 18 years

^b ^The best-fitting models are in bold font

We fit BMI trajectories with mixed effect models [26], specifying fixed effects of each fractional polynomial term, reflecting the population-average trend, and random effects of each term per child, modeling the deviation of each child from the population-average. We applied a two-stage method [27] to select optimal mean and residual variance-covariance structures: first we used the most complex mean structure (m = 8, the model with all 8 candidate powers) to select the best variance-covariance structure from 8 candidates (autoregressive, spatial power, compound symmetry, heterogeneous, toeplitz, heterogeneous toeplitz, unstructured, and variance components); and then fixed this best variance-covariance structure to select the best mean structure from the 219 candidate models mentioned above. We used the Bayesian information criterion (BIC) [28] to make this selection.

We calculated individual-specific BMI trajectories by combining the estimated fixed effects, which are shared by all subjects within sex, with the predicted random effects, which are specific to each individual. This results in a unique predicted trajectory for each subject. To assess the goodness of fit for each individual BMI trajectory, we first calculated the residual between the observed BMI and the estimated individual-specific BMI trajectory, and then used these residuals to calculate the residual BMI variance for each child (note that a smaller value implies a better fit).

#### Step II

**Figure 1:**
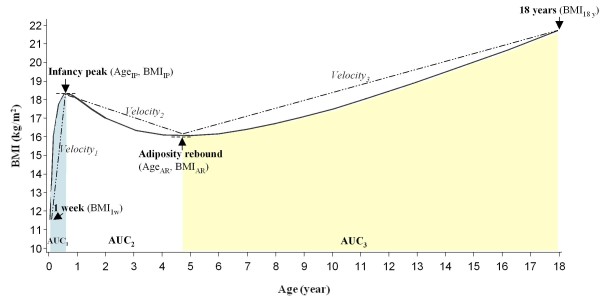
**Selected characteristics for the BMI trajectory of a hypothetical child**. Velocity_1 _between 1 week and infancy peak, Velocity_2 _between infancy peak to adiposity rebound, Velocity_3 _between adiposity rebound and 18 years of age. Area under curve (AUC_1_) between 1 week and infancy peak, AUC_2 _between infancy peak to adiposity rebound, AUC_3 _between adiposity rebound and 18 years of age. Note that the AUC below BMI value of 10 was not shown.

In this analysis, we were interested in ages and BMI values at two BMI trajectory milestones: infancy peak and adiposity rebound. We also estimated several other BMI trajectory characteristics related to these milestones, including age difference, change in BMI, velocity (linear rate of change in BMI), and area under curve (AUC) from 1 week to infancy peak, from infancy peak to adiposity rebound, and from adiposity rebound to 18 years of age. Figure 1 shows the key characteristics of BMI trajectory for a hypothetical child.

Based on the reported means and standard deviations (SD) of BMI trajectory milestones, or turning points on BMI curves, in the existing literature [14,17], we defined their hypothetical age intervals as within 3 SD of from the mean: 3 to 17 months for infancy peak and 15 months to 9.5 years for adiposity rebound. Because of the relatively small sample size in previous studies, we combined both sexes for these age intervals, to assure a large probability of identifying plausible BMI milestones. Then we divided age from 1 week to 18 years into 8,632 evenly spaced "minor" points 0.025 months (about 1 day) apart. We then estimated the velocity at each of these points by taking the first derivative of the individual-specific BMI trajectory curve. The criteria for existence of a milestone within the corresponding age interval were that two consecutive minor age points had opposite signs of the first derivative [14]: for infancy peak, the derivative at minor point k > 0 and point k + 1 < 0; for adiposity rebound, derivative at k < 0 and at k + 1 > 0. Within each pair of consecutive ages meeting the criteria above, the minor point with derivative closer to zero was designated the age at the milestone. Note that some children did not have both BMI milestones: infancy peak did not exist for 2 girls, while adiposity rebound did not exist for 37 boys and 62 girls. This occurs when the individual-specific curves lack a local maximum (infancy peak) or a local minimum (adiposity rebound) in the specified age ranges.

The predicted BMI (i.e., the point on the curve) at the minor age point identified is the basis for our BMI trajectory measures. We calculated the linear BMI velocity (defined as 'difference in BMI/difference in age') for three time periods: between 1 week of age and infancy peak, between infancy peak and adiposity rebound, and between adiposity rebound and 18 years of age. If BMI values at 1 week and 18 years of age were not observed at well-child visits, they were estimated from the fit individual-specific BMI trajectory models instead. The area under curve was estimated as the definite integral between the two age points. The SAS code used in Step II is available upon request.

#### Step III

We calculated pairwise Pearson correlation among pairs of BMI trajectory characteristics. Multivariable linear regression was used to examine predictors of the BMI trajectory characteristics; predictors included the child's sex, race/ethnicity, year of birth, z-score of birth weight, and the type of health insurance. Modeling was performed within a sub-sample with complete data on all these predictors.

## Results

### Sample characteristics

Table 1 shows characteristics of the analytic sample. Among the 3,289 children, 51.1% were boys; 71.8% non-Hispanic whites, 6.5% non-Hispanic blacks, 5.1% other race/ethnicity and 15.3% unknown race/ethnicity; 48.4% were born after 1990; the mean number of visits was 25 (range, 18 to 93). Among the total of 81,550 visits, over half occurred before 6 years of age.

### Models for BMI trajectory

**Figure 2:**
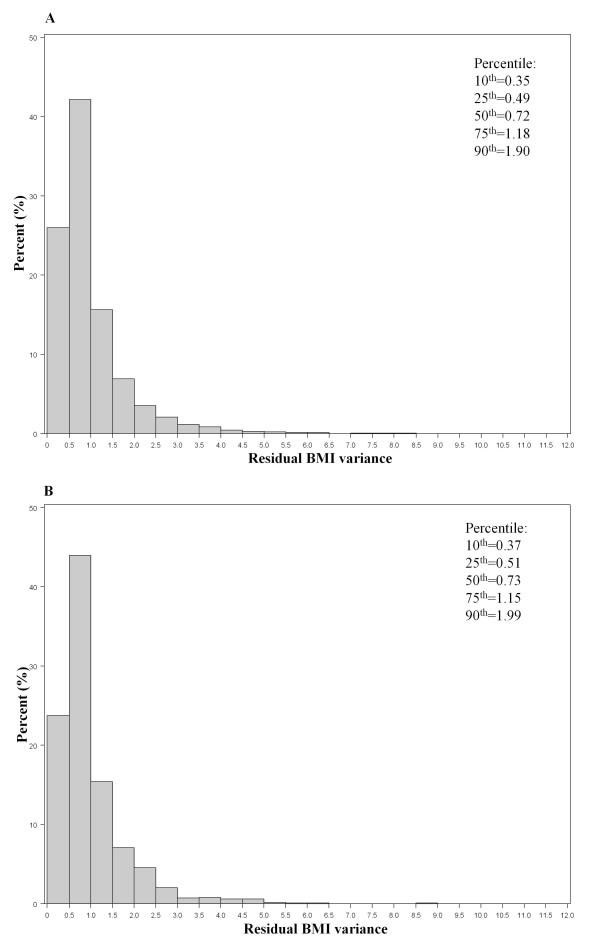
**Distribution of residual BMI variance (a measure for goodness of fit) among 3,289 children from 1 week to 18 years of age**. Lower residual BMI variance indicates a better fit of an individual's data points to the individual-specific model. A) Among 1,680 boys, B) Among 1,609 girls.

**Figure 3:**
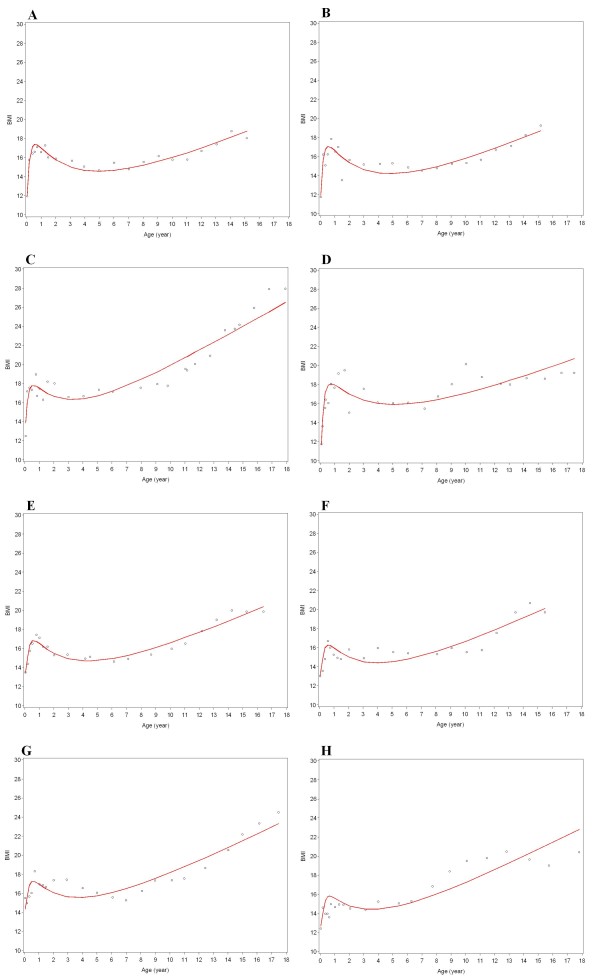
**Fitted BMI trajectories of 8 randomly selected children, one from each quartile of residual BMI variance**. Lower residual BMI variance indicates a better fit of an individual's data points to the individual-specific model. A) 1^st ^quartile - a boy (residual BMI variance = 0.21), B) 2^nd ^quartile - a boy (0.60), C) 3^rd ^quartile - a boy (1.08), D) 4^th ^quartile - a boy (1.26), E) 1^st ^quartile - a girl (0.23), F) 2^nd ^quartile - a girl (0.69), G) 3^rd ^quartile - a girl (0.85), H) 4^th ^quartile - a girl (1.59).

Among the 8 candidate variance-covariance structures, the autoregressive structure had the lowest BIC in the model 8 candidate polynomials, and was thus chosen for further selection of the best mean structure from the 219 candidate models. The mean of BIC values of these candidate models was 168,029 (SD, 7,841) for boys, and 158,490 (SD, 8,006) for girls. Table 2 shows goodness of fit for the best models by degree. For boys, the best model (lowest BIC) was "BMI = 96.8 - 3.6*Age^(-2) ^+ 51.0*Age^(-1) ^- 134.9*Age^(-0.5) ^- 24.4*ln(Age) + 4.6*Age^0.5^, and for girls it was "BMI = 90.8 - 3.2*Age^(-2) ^+ 47.0*Age^(-1) ^- 125.4*Age^(-0.5) ^- 22.7*ln(Age) + 4.3*Age^0.5^. Overall, these two 5^th^-degree models fit BMI trajectories of most children with reasonable accuracy, according to the distribution of residual BMI variances: inter-quartile range 0.49-1.18 BMI units for boys and 0.51-1.15 for girls (Figure 2). Figure 3 shows observed BMI values and individual-specific fitted BMI trajectories of 8 children randomly selected within quartile of residual BMI variance by sex.

### BMI trajectory characteristics and their correlations

Table 3 shows means and medians of BMI trajectory characteristics. The mean age at infancy BMI peak was 7.2 months for boys and 7.4 months for girls; the mean BMI at infancy peak was 17.8 kg/m^2 ^for boys and 17.3 kg/m^2 ^for girls. The mean age at adiposity rebound was 49.2 months for boys and 46.8 months for girls; the mean BMI at adiposity rebound was 15.6 kg/m^2 ^for boys and 15.5 kg/m^2 ^for girls.

**Table 3:** Means and medians of childhood BMI trajectory characteristics, by sex

	Boys (N = 1,680)			Girls (N = 1,609)		
**BMI trajectory characteristics**	**n^a^**	**Mean (SD)**	**Median (range)**	**n^b^**	**Mean (SD)**	**Median (range)**

**1 week to infancy peak**						

Age at infancy peak, months	1680	7.2 (0.9)	7.1 (3.9, 12.5)	1607	7.4 (1.1)	7.3 (3.3, 14.2)

BMI at infancy peak, kg/m^2^	1680	17.8 (0.9)	17.7 (15.0, 20.6)	1607	17.3 (0.9)	17.2 (14.5, 20.3)

Change in BMI, kg/m^2^	1680	10.7 (5.4)	10.7 (-8.2, 30.8)	1607	8.5 (5.1)	8.2 (-12.6, 28.1)

Velocity, kg/m^2^/month	1680	1.58 (0.82)	1.57 (-1.31, 5.10)	1607	1.21 (0.75)	1.15 (-1.50, 5.65)

Area under curve (kg/m^2^-months)	1680	114 (18)	112 (60, 207)	1607	116 (21)	113 (45, 242)

**Infancy peak to adiposity rebound**						

Age at adiposity rebound, months	1643	49.2 (11.9)	50.0 (24.0, 84.2)	1547	46.8 (11.0)	47.1 (24.1, 85.3)

BMI at adiposity rebound, kg/m^2^	1643	15.6 (1.3)	15.5 (11.9, 19.9)	1547	15.5 (1.2)	15.4 (11.8, 19.4)

Age difference, months	1643	42.0 (12.0)	42.8 (14.2, 76.7)	1547	39.4 (11.2)	40.0 (14.0, 77.0)

Change in BMI, kg/m^2^	1643	-2.2 (0.8)	-2.2 (-4.7, -0.2)	1547	-1.8 (0.7)	-1.9 (-4.0, -0.2)

Velocity, kg/m^2^/month	1643	-0.05 (0.01)	-0.05 (-0.08, -0.01)	1547	-0.04 (0.01)	-0.05 (-0.08, -0.01)

Area under curve (kg/m^2^-months)	1643	680 (182)	690 (251, 1332)	1547	629 (170)	633 (239, 1301)

**Adiposity rebound to age 18 years**						

Change in BMI, kg/m^2^	1643	8.3 (3.0)	7.5 (2.9, 20.0)	1547	8.1 (2.6)	7.5 (2.6, 18.8)

Velocity, kg/m^2^/month	1643	0.02 (0.01)	0.02 (0.00, 0.05)	1547	0.02 (0.01)	0.02 (0.00, 0.05)

Area under curve (kg/m^2^-months)	1643	3206 (590)	3115 (2010, 4985)	1547	3213 (534)	3139 (2008, 4725)

BMI, body mass index; SD, standard deviation

^a ^37 boys had no adiposity rebound

^b ^2 girls had no infancy peak and 62 girls had no adiposity rebound

Table 4 shows pairwise correlations between BMI trajectory characteristics. For simplicity, we only report correlations among the total sample, because stratification analysis by child sex did not yield considerable differences. Overall, the within-period correlations were stronger than between-period correlations. Age at infancy peak was weakly inversely correlated with age at adiposity rebound (r = -0.09). BMI at infancy peak and at adiposity rebound were strongly positively correlated (r = 0.76). BMI velocity and AUC from 1 week to infancy peak were weakly correlated with those from infancy peak to adiposity rebound (r = -0.27 for velocity, r = 0.01 for AUC) and with those from adiposity rebound to age 18 years (r = -0.02 for velocity, r = 0.28 for AUC). In contrast, BMI velocity (r = 0.40) and AUC (r = -0.87) from infancy peak to adiposity rebound were moderately or strongly correlated with those from adiposity rebound to age 18 years.

**Table 4:** Correlation matrix of childhood BMI trajectory characteristics (N = 3,289)

		1 week to infancy peak					Infancy peak to adiposity rebound						Adiposity rebound to age 18 years	
**Parameters**		**1**	**2**	**3**	**4**	**5**	**6**	**7**	**8**	**9**	**10**	**11**	**12**	**13**

**1 week to infancy peak**														

1	Age at infancy peak, months													

2	BMI at infancy peak, kg/m^2^	0.39												

3	Change in BMI, kg/m^2^	-0.03	0.45											

4	Velocity, kg/m^2^/month	-0.25	0.34	**0.96**										

5	Area under curve (kg/m^2^-months)	**0.96**	**0.63**	0.07	-0.14									

**Infancy peak to adiposity rebound**														

6	Age at adiposity rebound, months	-0.09	0.15	0.12	0.11	-0.08								

7	BMI at adiposity rebound, kg/m^2^	**0.59**	**0.76**	0.26	0.13	**0.76**	**-0.48**							

8	Age difference, months	-0.17	0.12	0.13	0.13	-0.16	**0.99**	**-0.53**						

9	Change in BMI, kg/m^2^	0.46	0.01	-0.13	-0.20	0.42	**-0.91**	**0.66**	**-0.94**					

10	Velocity, kg/m^2^/month	**0.84**	0.23	-0.10	-0.27	**0.79**	**-0.54**	**0.71**	**-0.61**	**0.82**				

11	Area under curve (kg/m^2^-months)	-0.06	0.33	0.21	0.19	0.01	**0.98**	-0.32	**0.97**	**-0.87**	**-0.51**			

**Adiposity rebound to age 18 years**														

12	Change in BMI, kg/m^2^	-0.10	-0.18	-0.07	-0.01	-0.09	**-0.94**	0.38	**-0.93**	**0.79**	0.38	**-0.93**		

13	Velocity, kg/m^2^/month	-0.08	-0.18	-0.07	-0.02	-0.07	**-0.95**	0.39	**-0.93**	**0.80**	0.40	**-0.93**	**0.99**	

14	Area under curve (kg/m^2^-months)	0.22	0.16	0.03	0.00	0.28	**-0.94**	**0.71**	**-0.95**	**0.91**	**0.64**	**-0.87**	**0.91**	**0.92**

Strong correlations (|r| ≥ 0.5) are bold

### Predictors of BMI trajectory characteristics

Table 5 shows the adjusted associations between BMI trajectory characteristics and their predictors from multivariable linear regression models. On average, girls had older age and lower BMI at infancy peak, but younger age at adiposity rebound, than boys. Girls had smaller velocity from 1 week to infancy peak (increase). Girls had smaller velocity (decrease) and smaller AUC from infancy peak to adiposity rebound. Non-Hispanic blacks had younger age at adiposity rebound, smaller AUC from infancy peak to adiposity rebound, but greater AUC and velocity from adiposity rebound to 18 years of age, than non-Hispanic whites. Greater z-score of birth weight was associated with younger age at adiposity rebound; higher BMI at both infancy peak and adiposity rebound; smaller velocity from 1 week to infancy peak; greater AUC from 1 week to infancy peak and from adiposity rebound to 18 years of age. BMI trajectory characteristics did not differ considerably by the three intervals of birth year, 1979-1984, 1985-1989, and 1990-1994, or the two types of health insurance, Medicaid and non-Medicaid.

**Table 5:** Predictors of BMI trajectory characteristics, from multivariable linear regression models that include all covariates in the table

		Mean difference in the BMI trajectory characteristic (95% confidence interval)						
		**Girls(vs boys)**	**Race/ethnicity (vs white)**		**Year of birth (vs 1979 ~ 1984)**		**Z-score of birth weight**	**Medicaid (vs non-Medicaid)**
				
**BMI trajectory characteristics**	**n^a^**		**Black**	**Other**	**1985 ~ 1989**	**1990 ~ 1994**		

**1 week to infancy peak**								

Age at infancy peak, months	2128	0.2 (0.2, 0.3)	-0.1 (-0.2, 0.1)	-0.1 (-0.3, 0.0)	0.0 (-0.2, 0.1)	0.0 (-0.1, 0.2)	0.0 (0.0, 0.1)	0.1 (-0.1, 0.4)

BMI at infancy peak, kg/m^2^	2128	-0.5 (-0.6, -0.4)	0.0 (-0.2, 0.1)	0.0 (-0.1, 0.2)	0.2 (0.0, 0.3)	0.1 (0.0, 0.3)	0.2 (0.2, 0.3)	0.1 (-0.1, 0.3)

Change in BMI, kg/m^2^	2128	-2.2 (-2.6, -1.7)	-0.1 (-0.9, 0.6)	0.3 (-0.5, 1.0)	0.7 (0.1, 1.4)	0.2 (-0.5, 0.9)	-1.4 (-1.7, -1.2)	-0.3 (-1.4, 0.8)

Velocity, 10^-2 ^kg/m^2^/month	2128	-35.4 (-41.7, -29.1)	-0.5 (-12.6, 11.5)	7.1 (-4.9, 19.1)	12.9 (2.7, 23.2)	3.1 (-7.1, 13.2)	-21.4 (-24.6, -18.2)	-7.1 (-23.8, 9.5)

Area under curve (kg/m^2^-months)	2128	1 (0, 3)	0 (-3, 3)	-2 (-5, 1)	1 (-2, 3)	2 (-1, 4)	3 (2, 3)	3 (-1, 7)

**Infancy peak to adiposity rebound**								

Age at adiposity rebound, months	2063	-2.1 (-3.0, -1.1)	-3.3 (-5.3, -1.3)	-1.6 (-3.5, 0.3)	0.1 (-1.5, 1.6)	-0.2 (-1.8, 1.4)	-0.6 (-1.1, -0.1)	-1.9 (-4.5, 0.8)

BMI at adiposity rebound, kg/m^2^	2063	-0.1 (-0.2, 0.0)	0.2 (0.0, 0.4)	0.0 (-0.2, 0.2)	0.2 (0.0, 0.3)	0.2 (0.0, 0.3)	0.3 (0.3, 0.4)	0.3 (0.0, 0.6)

Age difference, months	2063	-2.3 (-3.3, -1.3)	-3.2 (-5.2, -1.2)	-1.4 (-3.4, 0.5)	0.1 (-1.5, 1.7)	-0.2 (-1.8, 1.4)	-0.6 (-1.1, -0.1)	-2.0 (-4.7, 0.7)

Change in BMI, kg/m^2^	2063	0.3 (0.3, 0.4)	0.2 (0.0, 0.3)	0.0 (-0.1, 0.2)	0.0 (-0.1, 0.1)	0.0 (-0.1, 0.1)	0.1 (0.0, 0.1)	0.2 (0.0, 0.4)

Velocity, 10^-2 ^kg/m^2^/month	2063	0.6 (0.5, 0.7)	0.1 (-0.1, 0.2)	-0.1 (-0.2, 0.1)	0.0 (-0.2, 0.1)	0.0 (-0.1, 0.2)	0.1 (0.0, 0.1)	0.2 (0.0, 0.4)

Area under curve (kg/m^2^-months)	2063	-46 (-61, -31)	-48 (-78, -18)	-25 (-53, 4)	7 (-17, 31)	1 (-23, 25)	1 (-7, 9)	-24 (-65, 16)

**Adiposity rebound to age 18 years**								

Change in BMI, kg/m^2^	2063	-0.3 (-0.5, 0.0)	0.9 (0.4, 1.4)	0.4 (0.0, 0.9)	0.1 (-0.3, 0.5)	0.1 (-0.3, 0.5)	0.1 (0.0, 0.2)	0.4 (-0.2, 1.1)

Velocity, 10^-2 ^kg/m^2^/month	2063	-0.1 (-0.1, 0.0)	0.3 (0.1, 0.4)	0.1 (0.0, 0.3)	0.0 (-0.1, 0.1)	0.0 (-0.1, 0.1)	0.0 (0.0, 0.1)	0.1 (-0.1, 0.3)

Area under curve (kg/m^2^-months)	2063	-6 (-55, 42)	162 (65, 258)	70 (-23, 163)	32 (-47, 110)	44 (-34, 121)	70 (45, 94)	115 (-15, 246)

^a ^Sample size reduced due to missing data on race/ethnicity (15.3%) and birth weight (22.7%)

## Discussion

Using repeated growth measures from well-child visits, we fit childhood BMI trajectory from 1 week to 18 years of age and estimated BMI trajectory milestones and related characteristics. The majority of BMI trajectory characteristics were correlated with each other. Some BMI trajectory characteristics, including age and BMI at infancy peak and adiposity rebound, varied substantially by children's sex, race/ethnicity, and z-score of birth weight, but there was little evidence of cohort effects.

### BMI trajectory characteristics

We were able to estimate infancy BMI peak and adiposity rebound for most children. To the best of our knowledge, the present study is the first one to propose the period-specific AUC to characterize childhood BMI trajectory. We think this novel measure can reflect the child's cumulative "exposure" to excessive body weight; and its potential role in predicting later obesity and obesity-related diseases warrants further research.

One important but unanswered question in BMI trajectory literature is the extent of correlations among BMI trajectory milestones [14]. Our analysis showed that the majority of BMI trajectory characteristics were moderately or strongly correlated with each other. These correlations may be driven by 2 distinct biological forces. First, human growth is an inherently continuous process: the higher BMI is at infancy peak, the higher it will be at adiposity rebound. Second, the force of 'regression to mean' inhibits too extreme growth: the greater the velocity from 1 week to infancy peak, the lower the velocity from infancy peak to adiposity rebound. This multicollinearity can pose a challenge for separating the independent effects of these BMI trajectory characteristics on adult outcomes. However, the magnitude of correlations between BMI trajectory characteristics estimated in our study should be interpreted cautiously, because we did not observe the characteristics directly, but estimated these characteristics from the same fitted BMI trajectory.

In our cohort, boys and girls had different BMI trajectories and best-fitting models. In line with a previous study [14] and CDC 2000 growth charts, we found that girls were older and had lower BMI at infancy peak, and earlier adiposity rebound. These sex differences may be explained by genetics, growth or sexual hormones, diet, or physical activity levels. One of our novel findings is the racial/ethnic-differences in BMI trajectory characteristics. Compared to their white peers, non-Hispanic black children had BMI trajectory profiles that may be associated with higher risk of later obesity, including younger age at adiposity rebound [17], and larger velocity and greater AUC from adiposity rebound to 18 years of age. However, these racial differences should be interpreted with caution, given insufficient control of socio-economic status other than the type of health insurance. Consistent with the literature [14], we found that birth weight was a strong predictor for most BMI trajectory characteristics. Overall there were no substantial changes in BMI trajectory characteristics with year of birth, after controlling for other socio-demographics and z-score of birth weight. This suggests that childhood BMI trajectory was fairly stable across the analyzed years in our cohort.

### Modeling childhood BMI trajectory

Generally, there are two broad types of methods to estimate childhood BMI trajectory milestones: visualization and modeling [29]. Simple visualization was first used in early studies to determine adiposity rebound as the visual nadir or the point with the lowest BMI [30-32]. Although straightforward and convenient, the age at adiposity rebound estimated by simple visualization is quite arbitrary, especially for children with a flat valley around the nadir, and thus subject to large inter-observer variation.

Instead, several recent studies [14,17,18,33-36] have used statistical modeling to identify BMI trajectory milestones more objectively. Commonly, researchers select reasonable combinations of polynomial age terms to fit ordinary regression models within each child [17,18,35], or mixed effect models [14,33,34] among a group of children. Ordinary regression models require many data points for each child; their estimates are unbiased, but are often subject to large variability. In contrast, mixed effect models need fewer data points for each child and yield more stable estimates, although the estimates may be a little biased, especially for those with very few data points. A study comparing simple regression with mixed effect model for the same sample [36] found estimated BMI values at adiposity rebound were similar between them but estimated ages at adiposity rebound differed.

One common limitation of the existing studies [14,17] is that they only modeled a segment of childhood. Our novel contribution is developing a good parametric model for BMI trajectory throughout childhood, from 1 week to 18 years of age. Alternatively, some researchers use semi-parametric modeling [14,37], such as cubic and linear spline models, to fit childhood BMI trajectory. Cubic spline models are more flexible and thus may fit the data better than our fractional polynomial models, but they require arbitrary decisions on the number and locations of age 'knots', carry the potential for undesirable multiple infancy peaks and adiposity rebound points, and have limited generablizability of their fitted models due to heavy data-dependence [38,39]. Taken together, all current methods have both advantages and disadvantages. Our method can meet the high need of accurate milestone estimates and is flexible for various study populations and data structures, including missing data and non-fixed age of follow-ups; but it requires a large enough sample to build stable mixed effect models and strong statistical skills. We also note that, although the overall best-fitting fractional polynomial function for the total sample is not necessarily optimal for each individual, it is robust and appropriate especially for those children with only a few repeated BMI measures.

### Study strengths

Our study has several strengths. First, the large original dataset yielded a large analytic sample that met our strict eligibility criteria. Second, the small individual-level residual BMI variance supported the applicability of our selected fractional models for most children. Third, our methods can help researchers estimate novel BMI trajectory characteristics conveniently with common statistical software (e.g. SAS, R, and STATA). As a next step, we plan to develop user-friendly software to make our modeling and estimating process more convenient for general researchers and clinicians.

### Study limitations

Our study also had several limitations. One limitation is the quality of the clinical weight and height measures, although the use of a written protocol, annual scale calibration, periodic quality assurance, and mathematical correction for error in length measures under 2 years of age likely reduced measurement errors. In addition, we included only a small proportion of the total sample in the final analysis, and this sample seemed to differ from the excluded sample in race/ethnicity and type of health insurance. The over-representation of white children in the analytic sample makes our estimated BMI trajectory characteristics and possibly the best-fitting models less generalizable to racial/ethnic minorities. Our study population was from one multi-site pediatric practice in eastern Massachusetts. We did not validate our best-fitting models in an external population. Thus our *best-fitting models and estimated means and SD *for BMI trajectory characteristics may not be generalizable to other populations. But our *methods *for modeling childhood BMI trajectory and estimating BMI trajectory characteristics can be broadly used in other studies. Therefore, we recommend other researchers first select the best-fitting models for BMI trajectories in their own samples, and then estimate the corresponding BMI trajectory characteristics, rather than use our best-fitting model and estimated coefficients. Finally, our estimated associations between BMI trajectory characteristics and their predictors from multivariable regression models might be biased, as we did not adjust for some important potential confounders, such as parents' weight and height as well as family socio-economic status (except the type of child health insurance).

## Conclusions

Our mixed effect models with fractional polynomial functions fit childhood BMI trajectories well for most children seen at well-child visits in this sample. Using our method, one can conveniently estimate BMI trajectory milestones and related characteristics with reasonable accuracy. Future research should evaluate the independent and interactive roles of these novel BMI characteristics on later outcomes. Moreover, prenatal and early-life determinants of these BMI trajectory characteristics also warrant further investigation.

## Abbreviations

BMI: Body mass index; CI: Confidence interval; SD: Standard deviation; CENTURY: Collecting Electronic Nutrition Trajectory Data Using e-Records of Youth; HVMA: Harvard Vanguard Medical Associates; AUC: Area under curve.

## Competing interests

The authors declare that they have no competing interests.

## Authors' contributions

XW contributed to the development of study aims, led the analytic plan, conducted all data analyses, and wrote the manuscript. KK contributed to the development of study aims and the analytic plan, guided data analysis, and co-wrote the manuscript. MWG contributed to the development of study aims and the analytic plan; interpreted the results; and made major contributions to revising the manuscript. SLR prepared the dataset, checked SAS programming, and revised manuscript. EMT was the principal investigator of this study; and contributed to the development of the study aims and the analytic plan, result interpretation, and revision of the manuscript. All authors read and approved the final manuscript.

XW has full access to the data and takes responsibility for the integrity of the data and the accuracy of the data analysis.

## Pre-publication history

The pre-publication history for this paper can be accessed here:

http://www.biomedcentral.com/1471-2288/12/38/prepub
